# Effects of insomnia-focused psychological and behavioral interventions on depressive symptoms in children and adolescents: a systematic review and meta-analysis

**DOI:** 10.1007/s00787-026-03146-9

**Published:** 2026-08-06

**Authors:** Britta Galling, Jiamiao Wu, Amelie Oberstadt, Alexander Dück, Le Xiao, Alexander Prehn-Kristensen, Dirk Lehr

**Affiliations:** 1 Institute for Child and Adolescent Psychiatry, Center of Integrative Psychiatry GmbH, Christian-Albrecht University Kiel, Kiel, Germany; 2 Faculty of Social Work and Childhood Education, Hamburg University of Applied Sciences, Hamburg, Germany; 3 Key Laboratory of Intelligent Drug Research and Development for Mental Disorders, Beijing Anding Hospital, National Clinical Research Center for Mental Disorders, National Center for Mental Disorders, Capital Medical University, Beijing, China; 4 Department of Psychology, Faculty of Human Sciences, MSH Medical School Hamburg - University of Applied Sciences and Medical University, Hamburg, Germany; 5 Department of Child and Adolescent Psychiatry and Neurology of Rostock, University Medical Center, Rostock, Germany; 6 German Center for Child and Adolescent Health (DZKJ), Partner Site Greifswald/Rostock, Rostock, Germany; 7 Department of Health Psychology and Applied Biological Psychology, Faculty of Sustainability, Leuphana University, Lüneburg, Germany

**Keywords:** CBT-I, Depression, Digital health, Meta-analysis, Prevention, Sleep disorders, Children and adolescents

## Abstract

Psychological and behavioral interventions for insomnia are increasingly recognized as promising approaches not only to improve sleep but also to influence mental health outcomes in children and adolescents. This systematic review and meta-analysis examined the effects of insomnia-focused psychological and behavioral interventions on depressive symptoms, with particular attention to potential across selective and indicated prevention as well as adjunctive treatment contexts. A comprehensive literature search was conducted in PubMed/MEDLINE, PsycINFO, the Cochrane Library, and major Chinese databases (CNKI, Wanfang) from inception to December 30, 2025. Randomized controlled trials comparing psychological or behavioral insomnia interventions with control conditions and reporting changes in depressive symptoms in children and adolescents were included. Random-effects meta-analyses, meta-regression analyses, and within-arm standardized mean change analyses were performed. Eight studies comprising 904 participants (mean age=13.46 years) were identified. Across all studies, depressive symptoms improved from baseline to post-intervention, with a significant overall benefit of insomnia-focused interventions compared with control conditions (SMD=0.238, 95% CI=0.088–0.387, p=0.0019). Within-arm analyses indicated that effects in passive-comparator studies reflected genuine intervention benefit rather than spontaneous remission. Subgroup analyses showed no significant moderation by delivery format or setting, including digital interventions. The high intervention completion rate across trials (87%) supports the potential of insomnia as an indirect, less-stigmatizing entry point for depression prevention in children and adolescents — consistent with frameworks proposing that targeting comorbid, causally linked conditions may reduce depressive symptoms while lowering barriers to engagement. Integrating evidence-based insomnia interventions into clinical, school-based, and digital settings could substantially extend the reach of early mental health strategies.

**Supplementary Information:**

The online version contains supplementary material available at https://doi.org/10.1007/s00787-026-03146-9.

## Plain language summary

Many children and adolescents struggle with insomnia – persistent difficulties falling or staying asleep, or in younger children, refusal or struggles at bedtime – which can have wide-ranging effects on their mood, concentration, and overall wellbeing. Insomnia is known to be closely linked to behavioral depression, and children and adolescents with sleep problems are at increased risk of developing depressive symptoms. This raises an important question: Can treating insomnia help prevent or reduce depression? In this study, we systematically reviewed and statistically combined the results of eight randomized controlled trials involving over 900 children and adolescents. We found that psychological and behavioral treatments for insomnia – such as cognitive-behavioral therapy for insomnia (CBT-I) – led to meaningful reductions in depressive symptoms compared to control groups. Importantly, these benefits were observed across a range of settings, including clinics, schools, and digital platforms, and across different age groups. These findings suggest that addressing sleep problems in children and adolescents may be a promising and accessible way to also support their mental health. Because sleep-focused treatments tend to be brief, structured, and less stigmatizing than traditional mental health interventions, they may be particularly well suited as an early step in supporting at-risk children and adolescents.

**Supplementary Information:**

The online version contains supplementary material available at https://doi.org/10.1007/s00787-026-03146-9.

## Introduction

### Insomnia and depressive symptoms in children and adolescents

Sleep plays a foundational role in physiological, cognitive, and emotional functioning throughout life, but its importance is particularly pronounced during childhood and adolescence [1]. During these developmental periods, sleep supports brain maturation, synaptic refinement, and the establishment of regulatory systems underlying executive functioning and emotional control. Neuroscientific evidence highlights that adolescent sleep is closely tied to the maturation of key brain regions [2], making sleep disruption especially consequential for normative neurodevelopment and long-term mental health outcomes [3]. There is evidence that insufficient sleep during adolescence is associated with alterations in neural connectivity and emotional regulation circuits, underscoring sleep’s critical contribution to cognitive, affective, and behavioural stability [4].

Against this developmental backdrop, epidemiological studies indicate that insomnia and insomnia-related complaints are common during adolescence, although prevalence estimates vary depending on diagnostic criteria and assessment methods. Insomnia manifestations vary across developmental stages: in younger children, common presentations include bedtime refusal, prolonged sleep onset requiring parental presence, and frequent night wakings, whereas adolescents more typically report difficulties initiating sleep, early morning awakening, and non-restorative sleep. Population-based cross-sectional studies using structured diagnostic criteria (DSM-IV or ICSD-II, requiring symptoms on ≥ 3 nights per week for at least one month) report prevalence rates of 9–11% among adolescents [5–7], whereas broader estimates based on self-report questionnaires suggest that approximately 20–30% of adolescents experience clinically significant insomnia symptoms [8, 9]. In younger children (aged 5–12 years), population-based studies report insomnia symptom prevalence of approximately 20%, though data using formal diagnostic criteria in this age group remain limited [10]. Prevalence rates are even higher among children and adolescents with depressive symptoms or diagnosed depression [11].

Adolescence represents a particularly sensitive developmental window for sleep disturbances. Puberty is accompanied by a well-documented circadian shift (which frequently conflicts with early school start times and social demands) and a substantial reorganization of sleep architecture [12–14]. The maturational processes, combined with increasing psychosocial stressors and greater autonomy over bedtime behavior, render adolescents particularly vulnerable to the development of insomnia [1, 12].

Insomnia in children and adolescents is associated with a wide range of psychosocial and functional impairments, which may act as both antecedents and consequences of sleep disturbance. These include poor sleep hygiene and dysfunctional pre-sleep cognitions, impaired cognitive performance, heightened stress reactivity, emotional dysregulation, reduced health-related quality of life, and functional impairment. Importantly, insomnia is associated with an increased risk of psychopathology, particularly depressive symptoms [8, 9, 15]. Insomnia symptoms and their associated burden often persist across developmental stages, contributing to chronic sleep disturbance [16].

Consistent with this, a substantial proportion of children and adolescents with insomnia symptoms also meet criteria for psychiatric disorders. In a representative cross-sectional study, anxiety disorders, behavioral disorders, and mood disorders were reported in 33%, 23.9%, and 20.7% of children and adolescents with insomnia, respectively [17]. In clinical samples of adolescents presenting for insomnia evaluation, approximately three out of four fulfil criteria for at least one additional mental health condition [18]. Comparable proportions have been reported in administrative claims data, where roughly 75% of children and adolescents diagnosed with insomnia have comorbid psychiatric diagnoses, most commonly anxiety disorders, depression, or attention-deficit/hyperactivity disorder (ADHD) [19].

Despite this high degree of comorbidity, emerging evidence suggests that insomnia can also occur as an independent condition [20]. In particular, the conceptualisation of insomnia in the context of depression has shifted from viewing sleep disturbance solely as a symptom of psychopathology [21] to recognizing insomnia as a disorder with distinct etiological and maintenance mechanisms – a shift that is formally reflected in the DSM-5, which replaced the category of ‘primary insomnia’ with ‘insomnia disorder’ to underscore its diagnostic independence regardless of comorbid conditions [22]. Clinically, this distinction is important, as treatment approaches for sleep problems secondary to another disorder may differ from those for primary insomnia, highlighting the need for careful assessment and individualized intervention planning [23, 24].

Longitudinal studies and meta-analyses consistently demonstrate that insomnia often precedes the onset of depressive symptoms or depressive episodes, even after controlling for baseline depression [25, 26]. Individuals with insomnia have an approximately two- to threefold increased risk of developing depression compared to those without sleep problems, suggesting that insomnia can function as an independent risk factor for depression [27, 28]. Moreover, prospective cohort studies indicate that untreated insomnia not only precedes depression but may also contribute to depression recurrence and poorer treatment outcomes [28, 29].

At the same time, depressive symptoms can exacerbate and perpetuate sleep disturbances, indicating bidirectional maintenance processes between insomnia and depression [30].

### Insomnia as a modifiable target for the prevention and treatment of depression

The high prevalence rates of depression among children and adolescents and the often unsatisfactory outcomes of conventional treatments [31, 32] have increased interest in prevention and early intervention approaches. Their goal is to treat early in the developmental trajectory, potentially intercepting the onset or progression of depressive symptoms.

Given that insomnia represents a well-established risk factor for depression, focusing on insomnia may therefore contribute to the prevention of depression [33, 34]. This may include (i) universal approaches targeting entire populations (such as school-based programs), (ii) selective prevention approaches focussing on those with elevated risk (such as a family history of depression or exposure to significant stressors) as well as (iii) indicated prevention approaches addressing individuals with subclinical depressive symptoms and/or prodromal symptoms [35, 36].

Current recommendations for children and adolescents with insomnia consistently prioritize psychological and behavioral treatments, such as sleep hygiene education, bedtime routines, and behavioral interventions including cognitive-behavioral strategies as first-line approaches, with pharmacological options (such as melatonin) considered only in specific conditions (e.g., neurodevelopmental disorders such as autism spectrum disorder) or when behavioral measures have been fully optimized [37, 38]. Similar strategies have been applied when targeting insomnia in children and adolescents to prevent or reduce depressive symptoms: A landmark pilot trial augmenting standard depression treatment with cognitive behavioral therapy for insomnia (CBT-I) in adolescents demonstrated benefits for both sleep and depression outcomes [39]. This effect was confirmed by a review on the efficacy of CBT-I for the treatment of child and adolescent depression, demonstrating not only improvements in sleep but also reductions in depressive symptoms in most studies [40].

Psychological and behavioral insomnia interventions are particularly promising for several reasons: Addressing sleep problems directly may improve overall functioning and enhance the effectiveness of concurrent or subsequent depression-specific treatments, as unresolved insomnia can limit therapeutic progress. Moreover, psychological and behavioral interventions (such as CBT-I) are typically delivered in brief, structured, and standardised formats and have demonstrated robust efficacy [41]. These characteristics may reduce barriers to engagement and promote early success experiences, thereby enhancing motivation and self-efficacy. Moreover, there is increasing evidence supporting the effectiveness of digital CBT-I formats. An individual participant data meta-analysis in adults demonstrated that online insomnia interventions can reduce depressive symptoms [42], highlighting the potential of targeting insomnia as an indirect yet impactful strategy in depression care, that might also be successful in children and adolescents. More recently, a randomized clinical trial demonstrated that app-based CBT-I significantly reduced the incidence of major depressive disorder in children and adolescents with insomnia and subclinical depression [43], providing direct evidence for the preventive potential of digital insomnia interventions in younger populations. However, direct evidence for digital CBT-I formats specifically in children and adolescents remains limited, and further research in this population is warranted.

Taken together, these findings support a reciprocal and self-reinforcing relationship between insomnia and depression, underscoring the clinical importance of addressing sleep disturbances as both a preventive and therapeutic target [27, 44].

### Rationale and aims of the present meta-analysis

Despite the growing evidence base, an important research gap remains. Existing reviews of insomnia interventions in children and adolescents have predominantly focused on sleep outcomes, with depressive symptoms assessed inconsistently across studies. To the best of our knowledge, no meta-analysis to date has systematically synthesized evidence on the effects of insomnia-focused psychological and behavioral interventions on depressive symptom severity in this age group. Moreover, available randomized controlled trials vary considerably in terms of delivery setting (clinical, school-based, digital) and intervention format, and it remains unclear whether these contextual factors moderate treatment effects on depression [40].

Against this background, the aim of this systematic review and meta-analysis is to evaluate the effects of psychological and behavioral interventions for insomnia on depressive symptoms in children and adolescents with study-defined insomnia. Specifically, this review examines whether targeting insomnia is associated with reductions in depressive symptomatology. The focus on depressive symptoms - rather than anxiety or broader internalizing outcomes - reflects the comparatively stronger and more consistent longitudinal evidence linking insomnia to depression onset and recurrence, as well as the particular prevention gap in child and adolescent depression [25–27, 31, 32].

To structure the research question in a transparent and systematic manner, the PICO (Population, Intervention, Comparison, Outcome) framework was applied. Accordingly, the research question was formulated as follows: In children and adolescents with study-defined insomnia (Population), do psychological and behavioral interventions targeting insomnia (Intervention), compared with control conditions (care as usual, waitlist, or less intensive treatment) (Comparator), lead to a reduction in depressive symptoms (Outcome)?

## Methods

This systematic review, meta-analysis and meta-regression analysis followed the Preferred Reporting Items for Systematic reviews and Meta-Analyses (PRISMA 2020) guidelines to ensure a transparent, complete, and accurate report of methods and outcomes [45] and was prospectively registered with PROSPERO (registration number: CRD42024560366).

### Search strategy

At least two independent authors (KVL, AO, CS, JW, LX) searched PubMed/MEDLINE, PsycINFO, Cochrane Library, the International Clinical Trials Registry Platform (ICTRP), and Clinicaltrials.gov as well as China National Knowledge Infrastructure (CNKI) and WanFang (万方) without language restriction for randomized controlled trials (RCTs) comparing psychological or behavioral interventions for children and adolescents with a study-defined insomnia with a control condition, using combination of subject headings, and free text search terms (for details see Supplemental Tables 1 – Search terms). An initial search was conducted on June 17, 2024; a search update was performed on December 30, 2025 to capture recently published trials.

### Inclusion criteria

Inclusion criteria were (i) children and adolescents aged 6–17 years with (ii) a study-defined insomnia (or broader defined clinically relevant sleep problems), (iii) randomization to a psychological or behavioral intervention and a control condition, and (v) available meta-analyzable data on depressive symptom change (measured with a validated scale for depression). Studies were excluded if the primary focus was on primary sleep disorders other than insomnia (e.g., sleep-disordered breathing, restless legs syndrome, narcolepsy) or on delayed sleep-wake phase disorder; psychiatric or neurodevelopmental comorbidities were not grounds for exclusion.

The lower age limit of 6 years was chosen as validated depression outcome measures are generally normed for children aged 6 years and above. When studies also included subjects ≥ 18 years of age, study authors were contacted to request age-stratified data; if unavailable, studies were included if the mean sample age was ≤ 17 years. In line with the WHO definition of youth (15–24 years), the upper age limit was set at 24 years, representing a minor deviation from the registered protocol (≤ 21 years); the one affected study (Waite 2023) had a mean age of 16.9 ± 2.5 years, indicating that older participants represented a small minority.

### Risk of bias

The study quality of all included studies was evaluated using the Cochrane Risk of Bias (revised version, RoB 2) tool [46], a validated tool designed to assess the quality of randomised trials. Two reviewers (APK, AO) independently generated a ROB score. Here, discrepancies were resolved through consensus, and a third reviewer (BG) was consulted to resolve any remaining disagreements.

### Outcomes

The primary outcome of this meta-analysis was the reduction in depressive symptoms from baseline to study endpoint, measured with a validated scale for depression. Secondary outcomes were study-defined response of depression and study-defined remission of depression. 

### Data abstraction

All data from each study, including sample size, means and standard deviations for depressive symptom outcomes at baseline and post-intervention, and study and intervention characteristics required for subgroup and moderator analyses, were independently extracted in accordance with the Preferred Reporting PRISMA standard by two independent investigators (BG, AO, JW, LX); inconsistencies were resolved by consensus, with a third reviewer (BG) consulted where agreement could not be reached.

### Data synthesis and statistical analysis

We conducted a random effects [47] meta-analysis of outcomes for which ≥ 3 studies contribute data, using comprehensive meta-analysis V4 [48] and R studio for analyses and figures [49, 50]. In the primary analyses, the interventions and the control condition were compared at study-endpoint. Intent-to-treat (ITT) data were used whenever possible. Continuous outcomes were expressed as the standardized mean difference (SMD; Hedges’g [51]), with their 95% confidence intervals (CIs), calculated from post-intervention means and standard deviations of the intervention and control groups, as only post-intervention endpoint data were available across included studies. They were interpreted with reference to conventional benchmarks (small: 0.2, medium: 0.5, large: 0.8 [52]), acknowledging that their interpretation is context-dependent [53]. Where studies reported multiple depression scales, the scale most commonly used across studies was prioritized [54]. We explored the study heterogeneity using the chi-square test of homogeneity and *I*^2^ statistics, with *p* < 0.05 and *I*^2^ >50%, respectively, indicating significant heterogeneity. All analyses were two-tailed with alpha = 0.05.

Demographic information about the pooled study samples were calculated by weighting the study mean values according to sample size.

Where data allowed, we conducted exploratory maximum likelihood random effects meta-regression analyses of the primary outcome and the secondary outcomes to identify potential moderators, including (i) mean age, (ii) percentage of patients with depression at baseline, (iii) percentage of male participants, (iv) sample size, and (v) intervention duration. Baseline depression severity could not be compared in-between studies as heterogeneous scales were used. All meta-regression analyses were pre-specified, but should be interpreted with caution given the small number of included studies (k = 8). Moreover, we explored the following pre-specified subgroups for the primary outcome: (i) Comparator type (passive control [waitlist, treatment as usual without defined therapeutic content, e.g., routine clinical monitoring, medication management] vs. active control [e.g., sleep hygiene education, psychoeducation] for details of individual comparator conditions see Table 1), (ii) CBT-I (interventions were classified as CBT-I if they included at least two core CBT-I components (e.g., sleep restriction, stimulus control, cognitive restructuring, sleep hygiene, relaxation), in line with established CBT-I guidelines; for details on the components, see Table 1 ) [20, 55] vs. No CBT-I, (iii) delivery format (individual vs. group intervention), (iv) comparison of different age groups (teenager vs. children), (v) setting (clinical vs. school setting), (vi) delivery method (in-person vs. app), (vii) baseline depression (inclusion of patients with MDD diagnosis vs. no diagnosis).

**Table 1:** Insomnia intervention: study and sample characteristics

Study (Country), study identifier	*n* (analysis)	Age	Criteria for insomnia	Concurrent psychiatric illness	Depression severity at baseline	Prevention approach†	Interventions and intervention components/ comparators	Delivery method	Duration (wks)	Number of sessions/ frequencies	Assessments
INTERVENTIONS FOR PRESCHOOLERS AND YOUNG CHILDREN WITH INSOMNIA											
Hiscock 2015 (AUS) [56, 57], ISRCTN68819261	244 (ITT)	10.1yrs (5–12)	Behavioral sleep disorder: - Diagnostic label: ICDS-2 - Nightly criterion: sleep disturbances ≥ 3 nights/week - Duration threshold: ≥1 m	ADHD (100%), ASD (25%)	DASS = 38.2 ± 32.42	Selective prevention	Sleep consultation (“sleeping sound program”): - Educational: Sleep hygiene, sleep diary - Specific technique: Stimulus control, relaxation (customized to meet the child’s specific sleep needs)	In-person (patient and caregiver)	5	1 × 60 min (wk 1), 1 × 30 min (wk 3), f/u-call (wk 5)	BL, **3 m**, 6 m
							Passive comparator (Care as usual): - No routine assessment and management of sleep problems - Reviews of progress every six months, growth and blood - Prescribing of medications where required.	-	-	-	
Paine & Gradisar 2011 (AUS) [58], NR	42 (ITT/ LMM)	9.3yrs (7–13)	Behavioral Insomnia: - Diagnostic label: ICSD-2 - Nightly criterion: sleep disturbances ≥ 3 nights/week - Duration threshold: ≥1 m	-	SMFQ-P (NR)	Selective prevention	CBT for Behavioral Insomnia: - Educational: Sleep hygiene, sleep diary - Specific technique: Bedtime fading, sleep restriction, cognitive restructuring, relaxation	In-person (patient and caregiver)	8	6 × 45–60 min (wk 1, 2, 3, 4, 6, 8)	BL, **2 m**, 3 m, 8 m
							Passive comparator (waitlist): “A small number of WL participants reported engaging in specific activities during the waitlist period in an attempt to improve sleep.”	-	-	-	
INTERVENTIONS FOR TEENAGERS WITH INSOMNIA											
Blake 2016 (AUS) [59–63], ACTRN12612001177842	144 (mITT)	14.5yrs (12–17)	Sleep problems: - Cut-off: PSQI ≥ 5 (cut-off for clinically significant sleep disruptions)	MDD (50%), Anxiety (37%)	CES-D = 24.36 ± 9.31	Selective prevention	Sleep SENSE (CBT): - Educational: Sleep diary, sleep hygiene - Specific technique: Stimulus control, cognitive restructuring, third wave	In-person (group)	7	7 × 90 min	BL, **wk7**, 3 m, 6 m, booster, 2 yrs
							Active comparator (Study SENSE): Group study skills educational program, with sessions on personal organization, active listening, note-taking, memory, test-taking, critical reading, speech writing, and presentation.	In-person (group)	7	7 × 90 min	
Clarke 2015 (United States) [39], NCT00949689	41 (ITT/ LMM)	16.2yrs (12–20)	Chronic insomnia: - No formal diagnosis, but similar criteria used (DSISD) - Nightly criterion: sleep disturbance ≥ 3 nights/week - Duration threshold: ≥3 m	MDD (100%)	CDRS-*R* = 54.88 ± 9.12	Indicated prevention	CBT-D + CBT-I: - Educational: Sleep hygiene, sleep diary - Specific technique: Stimulus control, cognitive restructuring, relaxation	In-person	12	10x	BL, **wk12**, wk26
							Active comparator (CBT-D+Sleep hygiene)	In-person	12	10x	
Dewald-Kaufmann 2014 (Netherlands) [64], NR	60 (ITT/ LMM)	15.4yrs (12–19)	Chronic sleep reduction: - Cut-off: CSRQ ≥ 40 (chronic sleep deprivation)	-	CDI = 12.11 ± 4.73	Selective prevention	Gradual sleep extension and sleep hygiene: - Educational: Sleep hygiene, sleep diary - Specific technique: sleep extension	In-person	3	1x (telephone call)	BL, **wk3**
							Passive comparator (no instructions): - No instructions about sleep - Actigraph wearing and sleep diaries	-	-	-	
Huizhen Li 2022 (China) [65], NR	69 (NR)	15.7 ± 0.6yrs (14–17)	Chronic insomnia: - Diagnostic label: DSM-V - Nightly criterion: ≥3 nights/week - Duration threshold: ≥3 m	MDD (100%)	HAM-D (NR)	Indicated prevention	CBT: - Educational: Sleep hygiene - Specific technique: Stimulus control, sleep restriction, cognitive restructuring, relaxation	In-person	12	NR	BL, **wk12**
							Passive comparator (treatment as usual): - Inpatient clinical monitoring - Medication counseling and treatment (SSRIs and hypnotics)	-	-	-	
Waite 2023 (England) [66], ISRCTN85601537	40 (ITT)	16.9 ± 2.5yrs (14–23)	At least moderate insomnia: - Cut-off: ISI total score ≥ 15	UHR (100%)	DASS-21-dep = 29.02 ± 10.53	Selective prevention	SleepWell (CBT-based) + TAU: - Educational: Sleep hygiene, sleep diary - Specific technique: Stimulus control, cognitive restructuring, constructive worry, relaxation	In-person	12	8 × 60 min	BL, **3 m**, 9 m
							Passive comparator (usual care): - Infrequent contact with a general practitioner/ mental health professional for assessment, and prescription of psychotropic medication (mostly antidepressant medication) as needed	-	-	-	
Werner-Seidler 2023 (AUS) [67, 68], ACTRN12619001462178	264 (ITT)	14.7yrs (12–16)	At least mild insomnia: - Cut-off: ISI total score ≥ 10	MDD (13.75%) ANX (11%)	PHQ-A = 13.75 ± 0.51	Selective prevention	App-delivered CBT-I (“sleep ninja”): - Educational: Sleep hygiene - Specific technique: Stimulus control, cognitive restructuring	App-delivered	6	6 × 5–10 min	BL, **wk6**, wk14
							Active comparator (SMS sleep advice): - Weekly text messages for 6 weeks, including tips and suggestions to improve sleep taken from Sleep Ninja app.	SMS	6	6 SMS	

Assessments=primary endpoints bolded

†Prevention approach refers to the intended level of depression prevention with respect to depressive symptoms: selective prevention targets individuals at elevated risk by virtue of a comorbid sleep disorder (insomnia serving as the primary risk marker); indicated prevention targets individuals with already manifest (sub)clinical depressive symptoms (here: formal MDD diagnosis as inclusion criterion)

*ACTRN* Australian New Zealand Clinical Trial Registry registration number, *ADHD* attention-deficit/hyperactivity disorder, *anx *anxiety, *ASD *autism spectrum disorder, *AUS* Australia, *BL *baseline, *cc *control condition, *CBT-D *Cognitive Behavioral Therapy for Depression, *CBT-I *Cognitive Behavioral Therapy for Insomnia, *CDI *Children’s Depression Inventory, *CDRS-R *Children’s Depression Rating Scale–Revised (≥ 40 = clinical cut-off of likely depression), *CES-D *Center of Epidemiologic Studies Depression Scale (≥ 16 = possible clinical depression; ≥27 = high depressive symptomatology), *CSRQ *Chronic Sleep Reduction Questionnaire, *DASS *Depression Anxiety Stress Scale/ DASS-21 = short version with 21 items (0–9 = normal; 10–13 = mild; 14–20 = moderate; 21–27 = severe; >28 = extremely severe), *DSISD *Duke Structured Interview for Sleep Disorders, *DSM *Diagnostic and Statistical Manual of Mental Disorders, *EP *endpoint, *GAD-7*   Generalized Anxiety Disorder Scale, *ITT *intent to treat analysis including all randomized participant, *ISI *Insomnia Severity Index, *ISRCTN *International Standard Randomised Controlled Trial Number, *LMM *linear mixed model (equivalent to hierarchical linear model with direct maximum likelihood estimation), *mITT*   modified intent-to-treat (here, excluding randomized non-attenders), *m *month(s, MDD=Major Depressive Disorder, min=minute(s), *n *number of participants (randomized), *NCT *National Clinical Trial, *NR*   not reported, *PHQ-9 *Patient Health Questionnaire (0–4 = minimal; 5–9 = mild; 10–14 = moderate; 15–19 = moderately severe; 20–27 = severe; Clinical cut-off ≥ 10), *PSQI *Pittsburgh Sleep Quality Index, *SENSE *Sleep and Education, learning New Skills Early, *SMFQ-P *Short mood and feelings questionnaire-parent version, *SMS *short message service (text messages), *TAU *treatment as usual, *UHR *ultra high risk for psychosis, *wk(s) * week(s), *yrs *year(s)

As a sensitivity analysis, we excluded studies using an active comparator (i.e., studies in which the control condition consisted of an active treatment rather than waitlist or treatment as usual without defined therapeutic content) to examine whether the primary effect estimate was driven by comparator heterogeneity. Additionally, to assess whether observed between-group effects were primarily attributable to improvement in the intervention arm or deterioration/stagnation in the control arm, we calculated within-arm standardized mean changes (SMC) separately for intervention and control conditions across all studies, using pre- to post-intervention change scores standardized by baseline standard deviations. These are reported in Table 4.

Finally, we inspected funnel plots and used Egger’s regression test [69] and the Duval and Tweedie’s trim and fill method [70] to quantify whether publication bias could have influenced the results.

## Results

### Search result

**Fig. 1:**
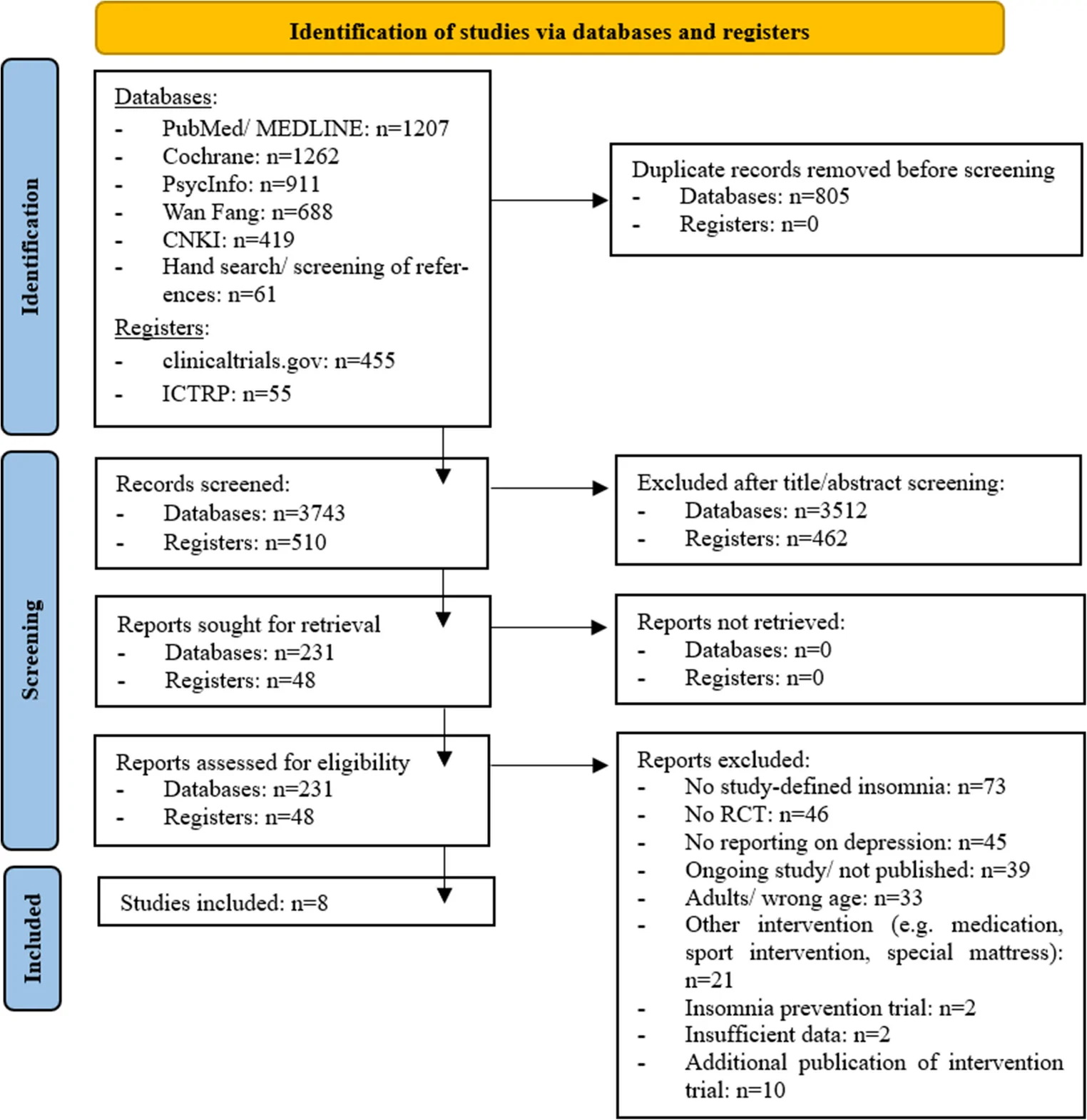
PRISMA diagram of the systematic literature search

The initial search yielded 4548 hits in databases (including 61 articles that were identified via screening previous reviews), and 510 hits in study regissters. After exclusion of duplicates (*n* = 805) and after evaluation on the title/abstract level, 279 full-text articles and/or study protocols were reviewed. Of those, 271 were excluded due to not fitting inclusion criteria, yielding 8 studies that were included in the meta-analysis (Fig. 1).

### Study, participant and intervention characteristics

Altogether, eight studies (n^randomized^=904, n^analyzed^=878) were included, with a mean sample size of 113 ± 93.5 (range = 40–244) [39, 56, 58, 59, 64–67, 71]. Across studies, the mean age was 13.46 years (range: 5–23), and 48.78% of participants were male. Two studies focused on children (*n* = 286; mean age = 9.98 years; range: 5–13), whereas six studies examined adolescents (*n* = 618; mean age = 15.08 years; range: 12–23). Most studies were conducted in Australia (four studies, *n* = 694) and Europe (two studies, *n* = 100). Interventions were delivered either in clinical settings (outpatients: five studies, *n* = 631, inpatients: one study, *n* = 69) or in a school setting involving students with insomnia symptoms (two studies, *n* = 204).

Across all studies, insomnia symptoms were reported by participants and/or their caregivers, although diagnostic and inclusion criteria varied slightly. Psychiatric comorbidities were common and included ADHD, autism spectrum disorder, anxiety, and depression.

Depressive symptoms were observed across all samples. Notably, two studies specifically included participants with both insomnia and a formal diagnosis of depression.

All interventions targeting preschoolers and young children were delivered in person and primarily involved caregivers. One intervention was predominantly educational, consisting of two in-person sessions and one follow-up call. The other evaluated a six-session CBT-I program.

Five of the six adolescent interventions were delivered in person. Four studies evaluated multi-session CBT-I programs (six to ten weekly sessions; see Table 1*–* for details) compared with treatment as usual or waitlist controls. One of these compared CBT-I added to CBT-D (cognitive behavioral treatment for depression) with CBT-D alone. Another study evaluated group-based CBT-I versus an educational program, and one tested a brief two-week sleep extension intervention. Only one study used a digital format: The CBT-I-app Sleep Ninja was compared with weekly text message–based sleep advice. Comparator conditions varied considerably: five studies used passive controls (TAU without defined treatment or waitlist), one augmented CBT-D with CBT-I (active comparator), one compared a digital CBT-I app with minimal active control (weekly SMS-based sleep advice), and one compared group CBT-I with a structured education program. These differences have implications for the interpretation of the pooled effect estimate, which primarily reflects the benefit of insomnia-focused intervention over no or minimal treatment for the majority of included studies; effect sizes from the three active-comparator studies should be interpreted as reflecting incremental rather than absolute treatment benefit.

### Risk of bias

Most domains demonstrated a low risk of bias, with only minimal concerns identified regarding the overall risk of bias. Bias was most frequently observed in the domains of outcome measurement and selection of reported results. The overall study attrition rate was very low, with all but 26 participants being included in the analysis through all studies (n^randomized^=904, n^analyzed^=878). Notably, Blake 2016 used a modified ITT approach excluding randomized non-attenders; while rated as overall low risk, this may slightly underestimate dropout-related bias compared to a strict ITT analysis (Table 2).

**Table 2:** Risk of bias assessments

Reference	Domains					Overall risk of bias
	Randomisation process	Deviations from intended interventions	Missing outcome data	Measurement of the outcome	Selection of the reported result	
Hiscock 2015 (AUS) [56, 57], ISRCTN68819261	Low	Low	Low	Low	Low	Low
Paine & Gradisar 2011(AUS) [58], NR	Some concerns	Low	Low	Low	Some concerns	Some concerns
Blake 2016 (AUS) [59–63], ACTRN12612001177842	Low	Low	Low	Low	Low	Low
Clarke 2015 (UnitedStates) [39], NCT00949689	Low	Low	Low	Low	Some concerns	Some concerns
Dewald-Kaufmann2014 (Netherlands) [64], NR	Low	Low	Low	Low	Some concerns	Some concerns
Huizhen Li 2022(China) [65], NR	Low	Low	Low	Some concerns	Low	Some concerns
Waite 2023 (England) [66], ISRCTN85601537	Some concerns	Low	Low	Some concerns	Low	Some concerns
Werner-Seidler 2023(AUS) [67, 68], ACTRN12619001462178	Low	Low	Low	Some concerns	Low	Some concerns

### Program acceptability

The interventions demonstrated a high level of overall acceptability, with 87.17% of participants completing the program across all intervention groups (range: 73.28–100%). The highest intervention drop-out rate (26.72%) was observed in the app-delivered CBT-I intervention. Notably, Sleep Ninja is a fully automated, self-guided smartphone application requiring no therapist involvement, making this completion rate particularly encouraging from a scalability perspective. An app-based intervention without therapist support that achieves a completion rate of 73.28% compares favorably with face-to-face CBT-I programs and underscores the potential of digital, low-resource formats for widespread deployment in children and adolescents mental health.

### Improvement of depressive symptoms

**Fig. 2:**
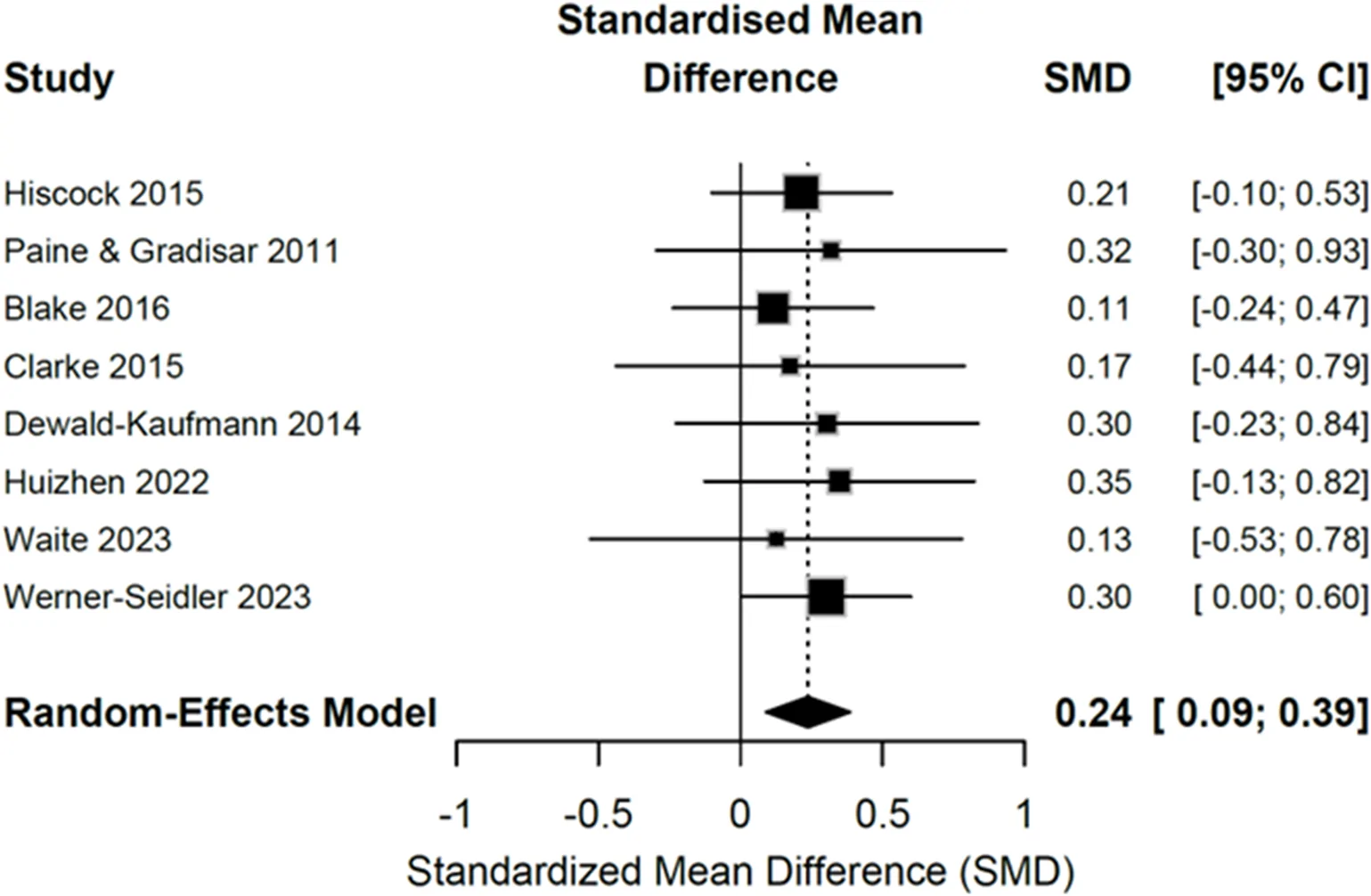
Improvement of depressive symptoms (forest plot). SMD=standardized meen difference (Hedges' g); CI=confidence interval. The size of each data point is proportional to the study samplesize. A positive SMD indicates greater reduction in depressive symptoms in the intervention group relative to the control group

Across all eight studies, insomnia-focused interventions were associated with a significant overall reduction in depressive symptoms compared with control conditions (SMD = 0.238, 95% CI [0.088, 0.387], *p* = 0.0019; see Fig. 2; for study-level details see Table 4).

Depression outcomes were assessed using a variety of validated instruments across the eight included studies: six studies used self-report measures (Depression Anxiety Stress Scale (DASS/ DASS-21) [56, 66]; Patient Health Questionnaire for Adolescents (PHQ-A) [67], Center for Epidemiologic Studies Depression Scale (CES-D) [59], Children’s Depression Inventory (CDI) [64], Hamilton Depression Rating Scale (HAMD) [65]), one study used a parent-rated scale (Short Mood and Feelings Questionnaire (parent version; SMFQ-P) [58]), and one study used a clinician-administered instrument (Children’s Depression Rating Scale – Revised (CDRS-R) [39]). The heterogeneity of outcome measures – differing in rating perspective, item content, and sensitivity to change – may affect the comparability of effect sizes across studies and should be considered when interpreting the pooled estimate. All analyses were based on endpoint data; no change scores were used. Seven studies used intent-to-treat analyses (four of which employed linear mixed models); one study used a modified intent-to-treat approach. No significant heterogeneity was observed (*I*^2^ = 0.0%, τ^2^ = 0, *p* = 0.992), though this finding should be interpreted cautiously given limited statistical power to detect heterogeneity with only eight included studies.

#### Subgroup-analysis

In subgroup analyses, no significant between-group differences were found across the seven predefined subgroup categories (all p for interactions > 0.05). The intervention remained consistently effective regardless of delivery format (app vs. in-person) or setting (clinical vs. school). The digital delivery format yielded a promising effect size (SMD = 0.301, *p* < 0.05) [67, 68], and the intervention showed particularly strong statistical evidence in participants without a baseline diagnosis of major depressive disorder (SMD = 0.229, *p* = 0.0062). However, several subgroup analyses were based on only one or two studies (e.g., app-based delivery, children as a separate age group); these findings should therefore be considered exploratory and interpreted with caution (Table 3).

**Table 3:** Exploratory subgroup analysis: effects on depressive symptoms

Subgroup category	Subgroup level	(k)	SMD (Hedges’ g)	95% CI	*P*-value	*P* for Interaction †
Comparator Type	Passive control	5	0.257	[0.048, 0.466]	0.0159	0.793
	Active control	3	0.217	[0.003, 0.431]	0.047	
CBT-I vs. No CBT-I	CBT-I	6	0.237	[0.058, 0.417]	0.0094	0.995
	No CBT-I	2	0.238	[-0.034, 0.511]	0.0862	
Delivery Format	Individual	6	0.253	[0.077, 0.429]	0.0049	0.746
	Group	2	0.198	[-0.086, 0.482]	0.1721	
Age Group	Teenagers (≥ 12 years)	6	0.238	[0.062, 0.415]	0.0082	0.992
	Children (5–13 years)	2	0.237	[-0.045, 0.518]	0.1002	
Setting	Clinical setting	6	0.260	[0.086, 0.434]	0.0033	0.617
	School setting	2	0.173	[-0.122, 0.468]	0.2499	
Delivery Mode	In-person	7	0.217	[0.044, 0.39]	0.0139	0.634
	App	1	0.301	[0.001, 0.601]	0.0492	
MDD diagnosis at baseline (inclusion criterion)	No diagnosis	6	0.229	[0.066, 0.329]	0.0062	0.797
	MDD	2	0.283	[-0.093, 0.659]	0.1406	

†P for interaction reflects the Q-test for between-subgroup heterogeneity, testing whether effect sizes differ significantly across subgroup categories

#### Meta-regression analysis

No significant moderating effects were found for age (*p* = 0.998), sample size (*p* = 0.942), percentage of male participants (*p* = 0.831), proportion of patients with depression at baseline (*p* = 0.701), du, or intervention duration (*p* = 0.901; for details see. However, the meta-regression analyses were underpowered to detect small to moderate moderator effects, and null findings should not be interpreted as evidence of absence of moderation.

#### Within-arm standardized mean changes (SMCs)

To allow transparent decomposition of the between-group effect sizes across heterogeneous comparator conditions, within-arm standardized mean changes (SMC) were calculated separately for intervention and control conditions (see Table 4). Across passive-comparator studies, control arms showed minimal improvement (SMC range: −0.16 to 0.50) or slight deterioration, suggesting that the observed between-group SMDs reflect genuine intervention benefit rather than spontaneous remission. In contrast, studies using active comparators (including one study augmenting CBT-D with CBT-I) showed substantial within-arm gains in both conditions, indicating that the effect sizes reflect incremental rather than absolute treatment benefit.

**Table 4:** Within-arm standardized mean changes (SMCs)

Study (country), study identifier	*n*	Interventions/ Comparators	Scale	Baseline: mean ± SD	Endpoint: mean ± SD	Standardized mean change (SMC)	Between-group SMD	Notes
Hiscock 2015 (AUS), [56, 57] ISRCTN68819261	244	Sleep consultation (“sleeping sound program”)	DASS	36.8 ± 25.3	29.4 ± 21.7	0.29	0.21	*Both arms improved; SMC_CON = 62% of SMC_INT*
		Passive comparator (Care as usual)		39.6 ± 27.8	34.7 ± 26.9	0.18		
Paine & Gradisar 2011 (AUS), [58] NR	42	CBT for Behavioral Insomnia	SMFQ-P	8.77 †	5.29 ± 1.12	0.64†	0.32	*†Author-reported within-group d used as SMC; 8.77 is ANCOVA-adjusted covariate*,* not raw mean*
		Passive comparator (Waitlist)		8.77 †	7.00 ± 1.21	0.38**†**		
Blake 2016 (AUS), [59–63] ACTRN12612001177842	144	Sleep SENSE (CBT)	CES-D	16.19 ± 9.80	13.65 ± 8.44	0.26	0.11	*Minimal change in both arms; very large SD at endpoint in control arm suggests high variability*
		Active comparator (Study SENSE)		15.32 ± 8.62	14.6 ± 18.01	0.08		
Clarke 2015 (United States), [39] NCT00949689	41	CBT-D + CBT-I	CDRS-R	54.2 ± 7.8	31.2 ± 9.7	2.95	0.17	*100% MDD; both arms show large within-arm gains - small between-group SMD reflects incremental augmentation benefit*
		Active comparator (CBT-D+Sleep hygiene)		55.6 ± 10.5	33.1 ± 11.6	2.14		
Dewald-Kaufmann 2014 (Netherlands), [64] NR	60	Gradual sleep extension and sleep hygiene	CDI	12.17 ± 4.53	11.18 ± 5.48	0.22	0.30	*Between-group SMD partly attributable to waitlist deterioration rather than intervention-driven improvement (control worsened slightly)*
		Passive comparator (no instructions)		12.04 ± 4.92	12.81 ± 5.05	**-**0.16		
Huizhen Li 2022 (China), [65] NR	69	CBT	HAMD	*NR*	10.61 ± 10.53	*NR*	0.35	*Baseline HAMD not reported in original paper; within-arm SMCs cannot be calculated*
		Passive comparator (treatment as usual)		*NR*	17.55 ± 11.84	*NR*		
Waite 2023 (England), [66] ISRCTN85601537	40	SleepWell (CBT-based) + TAU	DASS-21	30.4 ± 10.5	19.5 ± 13.4	1.04	0.13	*Substantial improvement in both arms; small between-group SMD*
		Passive comparator (usual care)		27.5 ± 12.4	21.3 ± 14.6	0.50		
Werner-Seidler 2023 (AUS), [67, 68] ACTRN12619001462178	264	App-delivered CBT-I (“sleep ninja”):	PHQ-A	13.82 ± 5.86‡	10.46 ± 5.90‡	0.57	0.30	*‡SE reported; SD estimated as SE×√n (n intervention = 132*,* n control = 133).*
		Active comparator (SMS sleep advice)		13.68 ± 5.88‡	12.33 ± 6.38‡	0.23		

*SMC* Standardized Mean Change ((Baseline Mean−Endpoint Mean)/ Baseline SD). Positive SMC indicates improvement (reduction in depression scores), while negative SMC indicates worsening

*SMD* standardized mean difference. Interpretation: Between-group SMDs may overestimate absolute treatment benefit when control arms deteriorate (e.g., Dewald-Kaufmann 2014: SMC Control Group=-0.16) and underestimate it when active comparators produce large within-arm gains (e.g., Clarke 2015: SMC Control = 2.14). Within-arm SMCs allow transparent decomposition of between-group effect sizes across heterogeneous comparator conditions

*ACTRN* Australian New Zealand Clinical Trial Registry registration number, *AUS *Australia, *CBT *cognitive-behavioral therapy, *CBT-D *cognitive-behavioral therapy for depression, *CBT-I *cognitive-behavioral therapy for insomnia, *CDI* Children’s Depression Inventory, *CDRS-R *Children’s Depression Rating Scale–Revised, *CES-D* Center for Epidemiologic Studies Depression Scale, *DASS *Depression Anxiety Stress Scale, *HAMD *Hamilton Depression Rating Scale, *ISRCTN *International Standard Randomised Controlled Trial Number, *NR*   not reported, *PHQ-A *Patient Health Questionnaire for Adolescents, *SMFQ-P* Short Mood and Feelings Questionnaire (parent version), *SENSE *Sleep and Education: learning New Skills Early, *SMS *short message service (text messages), *TAU *treatment as usual

#### Publication bias

Publication bias was evaluated using Egger’s and Begg’s tests, both of which yielded non-significant results (Egger’s intercept = 0.0158, 95%CI [-1.28, 1.31], *p* = 0.977; Kendall’s tau=-0.214, *p* = 0.548), indicating no substantial funnel plot asymmetry. The trim-and-fill procedure did not impute any missing studies, suggesting a symmetrical funnel plot and robust results.

### Study-defined response and remission

Data on depression response and remission were too limited to permit quantitative synthesis (response: k = 0; remission: k = 2). In Clarke 2015, remission rates ((CDRS-*R* < 28) were comparable between intervention (29%; 6/21) and control (35%; 7/20) participants [39]. In Werner-Seidler 2023, the proportion of participants with clinically significant depressive symptoms at endpoint (PHQ-A ≥ 10) was lower in the intervention group (45%; 33/74) than in the control group (64%; 68/106) [67], suggesting a meaningful reduction of symptomatic children and adolescents following the intervention.

## Discussion

This systematic review and meta-analysis examined whether psychological and behavioral interventions targeting insomnia in children and adolescents reduce depressive symptoms. Across eight randomized controlled trials involving 904 randomized participants, we found an improvement in depressive symptoms following insomnia-focused interventions compared to control groups (SMD = 0.238, 95% CI = 0.088–0.387, *p* = 0.0019). These findings support the conceptualization of insomnia as a potentially modifiable risk factor for depressive symptomatology in children and adolescents.

Interpreting the magnitude of this effect requires caution: as Cuijpers et al. [53] have argued, effect sizes in themselves cannot determine clinical relevance, as the outcome context and population characteristics must be considered. Notably, a tentative minimal important difference of SMD = 0.24 has been proposed for depression outcomes [53], and the present findings are broadly consistent with this threshold. Moreover, effect sizes in prevention studies are typically smaller than in treatment studies due to lower baseline severity, and even modest reductions in depressive symptoms at a population level may carry meaningful public health implications.

Notably, the effect of the psychological and behavioral interventions on depressive symptoms severity was not moderated by the proportion of depression diagnoses at baseline, suggesting intervention effects were potentially comparable across studies despite varying levels of diagnostic depression burden. This pattern is consistent with findings from adult samples, in which CBT-I has been shown to yield antidepressant effects across a range of baseline depression severity levels [42].

Several complementary mechanisms may explain our findings: First, insomnia represents a transdiagnostic process with neurobiological underpinnings in the development and maintenance of depressive symptoms - including dysregulation of the HPA axis, inflammatory pathways, and emotional memory consolidation during sleep, though direct evidence for these mechanisms in children and adolescents remains limited. Consistent with this, Werner-Seidler et al. [67] found in a mediation analysis that 59% of the total intervention effect on depressive symptoms was attributable to improvements in insomnia severity, providing direct mechanistic evidence from an adolescent sample. By targeting core sleep-related mechanisms, psychosocial insomnia interventions may improve emotional regulation, stress reactivity, and cognitive control across children and adolescents with varying proportions of major depressive disorder, leading to reductions in depressive symptoms regardless of baseline diagnostic burden.

A second hypthesis relates to a nonlinear relationship between sleep and depression. It is plausible that even subthreshold depressive symptoms may be sensitive to improvements in sleep, potentially allowing insomnia interventions to confer benefits before a manifest depressive disorder develops. In studies with higher proportions of depressive participants, sleep may still act as a particularly salient and modifiable factor, enabling comparable relative improvements. If supported, this hypothesis would suggest that insomnia interventions may be effective across multiple stages of prevention, including selective and indicated prevention approaches (universal prevention, targeting children and adolescents without pre-existing insomnia, has not yet been examined in this context).

Third, psychosocial insomnia interventions often incorporate therapeutic components that overlap with depression treatments, such as cognitive restructuring, behavioral activation - and may thereby improve depressive symptoms.

Component analyses of CBT-I in adults suggest that cognitive techniques targeting rumination and hyperarousal carry the primary antidepressant signal [72, 73]. And these same components have been identified as key active ingredients in CBT for depression children and adolescents [74], though data from the included studies were insufficient to examine component-level effects directly.

The observed effects emerged in diverse age groups (5–23 years), suggesting that these interventions are effective across different developmental stages, from childhood to young adulthood. Notably, some of the interventions included in this meta-analysis were tailored to a specific age group (for details see Table 1) and would not necessarily be directly transferable to other age ranges, which should be taken into account in the future development and implementation of interventions. For example, in adolescents (where insomnia frequently co-occurs with the physiological circadian phase delay of puberty), psychoeducation about this normative developmental process may itself carry protective effects by reducing the attribution of sleep difficulties to personal failure or psychopathology.

Notably, the observed effect size in our meta-analysis is smaller than those reported in adult samples where depression was the primary treatment target [42, 75–77]. These larger effects likely reflect greater baseline severity and the more direct overlap between insomnia treatment and depression treatment in clinical adult samples, compared with the more heterogeneous, non-clinical populations and preventive focus of the studies included here.

Overall, the present findings can be situated within the paradigm of indirect prevention and treatment as discussed by Cuijpers and Kazdin [33, 34]. It should be noted, however, that causal prevention claims remain inferential, as the included studies demonstrated symptom reduction rather than prevention of depressive disorder onset. Nevertheless, depressive symptoms might be targeted indirectly through the treatment of less stigmatized, comorbid condition that are causally linked to depression, such as insomnia. This approach may be particularly well-suited to children and adolescents, in whom stigma and a preference for self-directed care present substantial barriers to engagement with depression-specific services. The high program acceptability observed in this meta-analysis (87.17%) is consistent with this hypothesis.

All types of interventions, including educational programs, brief behavioral strategies, multi-session CBT-I, and digital CBT-I, were beneficial and clear differences could not be determined (which might be due to the limited number of studies). Regarding digital delivery, the single app-delivered intervention included in this meta-analysis (Sleep Ninja) yielded a preliminary effect size on depressive symptoms (SMD = 0.301, *p* < 0.05), comparable in magnitude to the overall effect. Given that this finding is based on one study, it should be interpreted with caution and considered preliminary. If replicated, it would be consistent with emerging adult data suggesting that digital formats can improve both insomnia and depression [78, 79]. It is also worth noting that many mobile health applications targeting sleep incorporate few validated CBT-I components beyond basic sleep hygiene [80], underscoring the importance of selecting theory-driven, rigorously evaluated tools. Given persistent barriers to help-seeking in children and adolescents [81, 82], digital insomnia interventions may represent a promising avenue for scalable early intervention, though larger trials are needed before firm conclusions can be drawn.

This meta-analysis has several strengths, including a comprehensive multi-database search (including Chinese databases), strict inclusion criteria, the exclusive use of randomized controlled trials, the inclusion of studies across a wide age range and varying settings, a low risk of bias across most domains, and minimal attrition rates. However, several limitations must be acknowledged: First, heterogeneity in insomnia definitions and diagnostic criteria may have contributed to variance in outcomes (however, the small number of included studies precluded a systematic examination of this effect); second, variability in depressive symptom measures may limit comparability across studies; third, predominance of studies from Australia and Europe may restrict generalization to other cultural contexts; fourth, limited number of long-term follow-ups prevents firm conclusions regarding sustained preventive or therapeutic effects; fifth, the small number of included studies precluded more detailed moderator analyses and substantially limited power to detect subgroup differences; sixth, the observed *I²*=0% should be interpreted cautiously, as with only eight included studies statistical power to detect heterogeneity is low, and substantial clinical heterogeneity across studies (in terms of age range, intervention format, depression measures, and diagnostic inclusion criteria) remains ; seventh, some studies included participants beyond the strict adolescent age range (Clarke 2015: range 12–20; Waite 2023: mean 16.9 ± 2.5 years, range 14–23); however, mean ages and standard deviations indicate that older participants represented a small minority in both studies; eighth, most depression outcomes relied on self-report measures, which may inflate effect sizes due to shared method variance with self-reported sleep outcomes; notably, two studies used non-self-report measures (parent-rated: Paine & Gradisar 2011; clinician-rated: Clarke 2015), which partially mitigates this concern; ninth, blinding of participants to behavioral interventions is inherently difficult, and expectancy effects cannot be excluded. Additionally, Embase and conference abstracts were not searched; however, we consider it unlikely that relevant studies were missed given the additional search of reference lists of included studies and reviews.

Despite these limitations, the present findings provide a promising basis for continued research and clinical translation. To advance this field, the following priorities are proposed: (1) future studies should establish standardized criteria for clinically meaningful improvement, including systematic reporting of depression response and remission rates alongside dimensional symptom scores; (2) head-to-head comparisons of CBT-I delivery formats in children and adolescents are needed [83, 84]; (3) component-level analyses should examine which CBT-I elements drive antidepressant effects; (4) longer follow-up periods are required to determine whether effects are sustained and whether treating insomnia in children and adolescents merely delays or genuinely prevents the onset of clinical depression; (5) studies should include transdiagnostic outcome measures such as global functioning and quality of life rather than restricting endpoints to diagnostic categories, to better capture the broad developmental benefits of sleep-focused interventions in children and adolescents.

Promising future directions also include the integration of insomnia-focused interventions into broader preventive or therapeutic frameworks for depression. Embedding CBT-I components within stepped-care or transdiagnostic treatment models may enhance overall treatment efficiency and address shared mechanisms underlying sleep and mood disturbances.

Given the developmental context, the role of caregivers warrants consideration. Future research should systematically examine the extent to which parental involvement, limit-setting, and sleep-related structure moderate treatment outcomes, as improvements in sleep may be constrained when environmental contingencies remain unchanged. Practical constraints such as shared bedrooms, irregular household routines, and parental shift work may limit the generalizability of CBT-I recommendations and should be considered in intervention design, particularly for families from lower socioeconomic backgrounds.

## Conclusion and clinical implications

Psychological and behavioral interventions for insomnia are associated with significant reductions in depressive symptoms in children and adolescents. While causal and preventive claims require further longitudinal evidence, the consistency of effects across diverse study designs and populatoins supports the potential value of insomnia-focused interventions within within selective and indicated prevention as well as adjunctive treatment frameworks. These findings suggest that insomnia a promising and modifiable target that warrants further investigation in the context of depression in children and adolescents.

From a clinical and public health perspective, integrating insomnia treatment into routine pediatric and child and adolescent mental health care may represent an important opportunity to improve children and adolescents mental health outcomes. In addition to traditional clinical settings, school-based and digitally delivered interventions may facilitate earlier identification and intervention, thereby extending the reach of evidence-based insomnia treatments. The observation of beneficial effects across both school-based and clinical settings, including children and adolescents with complex clinical profiles, highlights opportunities for broader implementation through school-based screening and intervention approaches as well as context-sensitive stepped-care models tailored to individual needs. Importantly, such strategies should also consider broader social risk factors, such as poverty and psychosocial adversity, which may interact with sleep disturbances and potentially limit the effectiveness of insomnia-focused interventions.

In this context, digital insomnia interventions hold promise for preventive approaches, as they enable early, low-threshold access to evidence-based treatment and may reduce barriers to care before depressive symptoms escalate. A key advantage of insomnia-focused interventions more broadly is that they target sleep rather than mental health directly, which may reduce perceived stigma - consistent with the indirect prevention and treatment paradigm described by Cuijpers and Kazdin [33, 34] - and increase acceptability among young people and their families. This indirect framing may facilitate engagement with evidence-based care at an earlier stage, making insomnia treatment a particularly suitable entry point within stepped-care models for children and adolescents at risk of depression. 

## Supplementary Information

Below is the link to the electronic supplementary material.

Supplementary Material 1

## Data Availability

The datasets generated and analyzed during the current study include aggregated data ex-tracted from published randomized controlled trials as well as additional data kindly provided by the original study authors. These data are available from the corresponding author upon reasonable request. No individual participant data was used. All data supporting the findings of this study are provided within the article and its supplementary materials.

## Declarations

### AI tools declaration

The authors used AI-based tools (Claude, Anthropic) to support the revision of the Introduction and Discussion sections. In addition, the entire manuscript was linguistically reviewed and refined using AI-based language editing tools. All AI-generated suggestions were reviewed, edited, and approved by the authors. No AI tool was used for data analysis, interpretation of results, or generation of conclusions.
