# Supplementary material for: Effects of insomnia-focused psychological and behavioral interventions on depressive symptoms in children and adolescents: a systematic review and meta-analysis
Source: Eur Child Adolesc Psychiatry. 2026 Aug 6;35(10):3099–116. doi: 10.1007/s00787-026-03146-9 (PMC13696559; doi:10.1007/s00787-026-03146-9)
Supplement: Supplementary file 1 [file 787_2026_3146_MOESM1_ESM.docx]

# Supplement

*Supplemental table 1 – Search terms*

| Database | Search Term |
| --- | --- |
| PubMed/ Medline | ("Sleep Initiation and Maintenance Disorders"[MeSH Terms] OR "sleep initiation and maintenance disorders"[All Fields] OR "insomnia"[All Fields] NOT "Insomnia, Fatal Familial"[MeSH Terms] OR "sleep initiation"[All Fields] OR "sleep maintenance"[All Fields] OR "sleeplessness"[All Fields] OR "sleep wake disorder"[All Fields]) AND ("child"[MeSH Terms] OR "child*"[All Fields] OR "adolescent"[MeSH Terms] OR "adolescent*"[All Fields] OR "infant"[MeSH Terms] OR "infan*"[All Fields] OR "pediatric"[All Fields] OR "paediatric"[All Fields] OR "toddler"[All Fields] OR "youth*"[All Fields] OR "school"[All Fields] OR "minors" [MeSH Terms] OR "minor*"[All Fields] OR "teenage*"[All Fields] OR "student*"[All Fields]) AND ("abdominal breathing"[All Fields] OR "autogenic training"[MeSH Terms] OR "autogenic training"[All Fields] OR "bedtime restriction"[All Fields] OR "behavior therapy"[MeSH Terms] OR "behaviour therapy"[All Fields] OR "behavior therapy"[All Fields] OR "behaviour intervention"[All Fields] OR "behavior intervention"[All Fields] OR "behavioral treatment" [All Fields] OR "behavioural treatment" [All Fields] OR "biofeedback"[All Fields] OR "biofeedback, psychology"[MeSH Terms] OR "biofeedback, psychology"[All Fields] OR "body monitoring" [All Fields] OR "BTII" [All Fields] OR "CBT-I" [All Fields] OR "CBT-Insomnia" [All Fields] OR "cognitive behaviour therapy"[All Fields] OR "cognitive behavior therapy"[All Fields] OR "cognitive behaviour therapies"[All Fields] OR "cognitive behavior therapies"[All Fields] OR "cognitive therapy"[All Fields] OR "counseling"[All Fields] OR "dCBT-I" [All Fields] OR “deep breathing”[All Fields] OR "desensitization relaxation" [All Fields] OR "eHealth" [All Fields] OR "family therapy"[All Fields] OR "guided imagery" [All Fields] OR "hypnosis"[All Fields] OR "iCBT-I" [All Fields] OR "imagery"[All Fields] OR "imagery training"[All Fields] OR „insomnia intervention“[All Fields] OR "internet-based" [All Fields] OR "mindfulness" [MeSH Terms] OR "mindfulness" [All Fields] OR "paradoxical intention"[All Fields] OR "physical activity"[All Fields] OR “progressive muscle relaxation”[All Fields] OR "psychoanaly*"[All Fields] OR "psychodynamic therapy"[All Fields] OR "psychotherapy"[MeSH Terms] OR "psychotherapy"[All Fields] OR "psychotherap*"[All Fields] OR "relaxation therapy"[MeSH Terms] OR "relaxation therapy"[All Fields] OR "relaxation therapies"[All Fields] OR "smartphone app" [All Fields] OR "smartphone application" [All Fields] OR "sleep education"[All Fields] OR "sleep hygiene"[MeSH Terms] OR "sleep hygiene"[All Fields] OR "sleep restriction"[All Fields] OR "sleep retraining"[All Fields] OR "sport intervention"[All Fields]  OR "stimulus control"[All Fields] OR "complementary therapies "[MeSH Terms]) AND ("1900/01/01"[PDAT] : "2024/06/17"[PDAT] NOT "Editorial"[Publication Type] NOT "Letter"[Publication Type] NOT "Comment"[Publication Type] NOT "Case Reports"[Publication Type] NOT "Biography"[Publication Type]) AND ("RCT" [All Fields] OR "randomized" [All Fields] OR "randomised" [All Fields] OR "clinical trial" [All Fields] OR "controlled trial" [All Fields]) |
| PsycInfo (via Ebsco) | („Insomnia“) OR („Sleep Wake Disorders") OR ("Sleep Quality") OR („Sleep Deprivation") OR MM(„Sleep Initiation and Maintenance Disorders“) OR („Sleep Initiation and Maintenance Disorders“) OR („sleep initiation“) OR („sleep maintenance“) OR („sleeplessness“) AND MM(„child“) OR („child“) OR MM(„adolescent*“) OR („adolescent*“) OR („infan*“) OR MM(„infant“) OR („infant“) OR („youth*“) OR MM(„minors“) OR („minors“) OR („minor*“) OR („school“) OR („student“) OR („Pediatrics“) OR („Child and Adolescent Psychiatrists") OR („Adolescent Psychiatry“) OR („Adolescent Psychology“) OR („Adolescent Psychopathology“) OR („Child Psychiatry“) OR („Child Psychology“) OR („Child Psychopathology") AND („abdominal breathing“) OR MM(„Autogenic Training“) OR („Autogenic Training“) OR („bedtime restriction“) OR („behaviour intervention“) OR („behavioral treatment“) OR („body monitoring“) OR MM(„biofeedback, psychology“) OR („biofeedback, psychology“) OR („Biofeedback Training“) OR („cognitive behaviour therap*“) OR MM(„Behavior Therapy“) OR („Behavior Therapy“) OR („cognitive therapy“) OR („Biofeedback“) OR („deep breathing“) OR („desensitization relaxation“) OR MM(„Relaxation Therapy“) OR („Relaxation Therapy“) OR („Cognitive Behavior Therapy") OR („Family Therapy") OR („Guided Imagery“) OR („imagery training“) OR („insomnia intervention“) OR („Imagery“) OR („Hypnotherapy“) OR („Hypnosis“) OR („Psychoanalysis“) OR („internet-based“) OR („Digital Interventions") OR („eHealth“) OR („Digital Mental Health Resources“) OR („Smartphone Use“) OR („Screen Time“) OR („smartphone app“) OR („smartphone application“) OR („sleep education“) OR MM(„sleep hygiene“) OR („sleep hygiene“) OR („sleep restriction“) OR („sleep retraining“) OR („sport intervention“) OR MM(„complementary therapies“) OR („complementary therapies“) OR MM(„Mindfulness“) OR („Mindfulness“) OR („Breathing Techniques") OR („paradoxical intention“) OR („Physical Activity") OR („Muscle Relaxation“) OR („psychoanaly*) OR („progressive muscle relaxation“) OR („Progressive Relaxation Therapy") OR („psychodynamic therapy“) OR („Psychodynamic Psychotherapy“) OR MM(„Psychotherapy") OR („Psychotherapy“) OR („psychotherap*“) OR („Relaxation") OR („Stimulus Control“) OR („CBT-I“) OR („BTII“) OR („CBT-Insomnia“) OR („Counseling“) OR („dCBT-I“) OR („iCBT-I“) AND (“RCT”) OR (“randomized”) OR (“randomised”) OR (“clinical trial”) OR (“controlled trial“) |
| Cochrane via Ovid | (("sleep initiation and maintenance disorders" or insomnia or "sleep initiation" or "sleep maintenance" or sleeplessness or "sleep wake disorder") and (child* or adolescent* or infan* or pediatric or paediatric or toddler or youth* or school or minor* or teenage* or student*) and ("abdominal breathing" or "autogenic training" or "bedtime restriction" or "behavior therapy" or "behaviour therapy" or "behaviour intervention" or "behavior intervention" or "behavioral treatment" or "behavioural treatment" or biofeedback or "biofeedback, psychology" or "body monitoring" or BTII or CBT-I or "CBT-Insomnia" or "cognitive behaviour therapy" or "cognitive behavior therapy" or "cognitive behaviour therapies" or "cognitive behavior therapies" or "cognitive therapy" or counseling or dCBT-I or "deep breathing" or "desensitization relaxation" or eHealth or "family therapy" or "guided imagery" or hypnosis or iCBT-I or imagery or "imagery training" or "insomnia intervention" or internet-based or mindfulness or "paradoxical intention" or "physical activity" or "progressive muscle relaxation" or psychoanaly* or "psychodynamic therapy" or psychotherapy or psychotherap* or "relaxation therapy" or "relaxation therapies" or "smartphone app" or "smartphone Application" or "sleep education" or "sleep hygiene" or "sleep restriction" or "sleep retraining" or "sport intervention" or "stimulus control" or "complementary therapies") and (RCT or randomized or randomised or "clinical trial" or "controlled trial")).af. |
| ICTRP | ("Sleep Initiation and Maintenance Disorders" OR "Insomnia" OR "Sleep Initiation" OR "Sleep Maintenance" OR "Sleeplessness" OR "Sleep Wake Disorder") AND ("abdominal breathing" OR "autogenic training" OR "bedtime restriction" OR "behavior therapy" OR "behaviour therapy" OR "behaviour intervention" OR "behavior intervention" OR "behavioral intervention" OR "behavioural intervention" OR "biofeedback" OR "biofeedback, psychology" OR "body monitoring" OR "BTII" OR "CBT-I" OR "CBT-Insomnia" OR "cognitive behavior therapy" OR "cognitive behaviour therapy" OR "cognitive behavior therapies" OR "cognitive behaviour therapies" OR "cognitive therapy" OR "counseling" OR "dCBT-I" OR "deep breathing" OR "desensitization relaxation" OR "eHealth" OR "family therapy" OR "guided imagery" OR "hypnosis" OR "iCBT-I" OR "imagery" OR "imagery training" OR "insomnia intervention" OR "internet-based" OR "mindfulness" OR "paradoxical intention" OR "physical activity" OR "progressive muscle relaxation" OR "psychoanaly*" OR "psychodynamic therapy" OR "psychotherapy" OR "psychotherap*" OR "relaxation therapy" OR "relaxation therapies" OR "smartphone app" OR "smartphone application" OR "sleep education" OR "sleep hygiene" OR "sleep restriction" OR "sleep retraining" OR "sport intervention" OR "stimulus control" OR "complementary therapies"), search in clinical trials in children only |
| Clinicaltrials.gov | Condition/ disease  "Sleep Initiation and Maintenance Disorders" OR "Insomnia" OR "Sleep Initiation" OR "Sleep Maintenance" OR "Sleeplessness" OR "Sleep Wake Disorder"  Other terms  "child" OR "adolescent" OR "infant" OR "pediatric" OR "toddler" OR "youth" OR "school" OR "minor*" OR "student"  Intervention/ treatment  "abdominal breathing" OR "autogenic training" OR "bedtime restriction" OR "behavior therapy" OR "behaviour therapy" OR "behaviour intervention" OR "behavior intervention" OR "behavioral intervention" OR "behavioural intervention" OR "biofeedback" OR "biofeedback, psychology" OR "body monitoring" OR "BTII" OR "CBT-I" OR "CBT-Insomnia" OR "cognitive behavior therapy" OR "cognitive behaviour therapy" OR "cognitive behavior therapies" OR "cognitive behaviour therapies" OR "cognitive therapy" OR "counseling" OR "dCBT-I" OR "deep breathing" OR "desensitization relaxation" OR "eHealth" OR "family therapy" OR "guided imagery" OR "hypnosis" OR "iCBT-I" OR "imagery" OR "imagery training" OR "insomnia intervention" OR "internet-based" OR "mindfulness" OR "paradoxical intention" OR "physical activity" OR "progressive muscle relaxation" OR "psychoanaly*" OR "psychodynamic therapy" OR "psychotherapy" OR "psychotherap*" OR "relaxation therapy" OR "relaxation therapies" OR "smartphone app" OR "smartphone application" OR "sleep education" OR "sleep hygiene" OR "sleep restriction" OR "sleep retraining" OR "sport intervention" OR "stimulus control" OR "complementary therapies" |
| 万方 (Wan Fang) | （主题: “未成年” OR “儿童” OR “青少年” OR “少儿” OR “幼儿” OR “小儿” OR “幼童” OR “儿科” OR“儿童科” OR “学校”） AND （主题: “失眠” OR “睡眠障碍” OR “入睡困难” OR “睡眠中断” OR “多梦” OR “眠浅” OR “睡眠维持时间” OR “夜啼” OR “入睡潜伏时间” OR “早醒” OR “睡眠质量”） AND ( 主题: “行为干预” OR “心理治疗” OR “行为治疗” OR “生物反馈” OR “认知行为” OR “认知行为” OR “心理咨询” OR “呼吸训练” OR “冥想” OR “正念” OR “肌肉” OR “太极” OR “心理动力” OR “睡眠教育” OR “睡眠卫生” OR “放松疗法” OR “数字疗法” OR “应用程序” OR “脱敏治疗” OR “家庭治疗” OR “精神分析” OR “中医” OR “中药” OR “按摩” OR “推拿” OR “针灸”）AND ( 主题: “随机对照” OR “随机”） |
| CNKI | （主题: “未成年” OR “儿童” OR “青少年” OR “少儿” OR “幼儿” OR “小儿” OR “幼童” OR “儿科” OR“儿童科” OR “学校”） AND （主题: “失眠” OR “睡眠障碍” OR “入睡困难” OR “睡眠中断” OR “多梦” OR “眠浅” OR “睡眠维持时间” OR “夜啼” OR “入睡潜伏时间” OR “早醒” OR “睡眠质量”） AND (主题: “行为干预” OR “心理治疗” OR “行为治疗” OR “生物反馈” OR “认知行为” OR “认知行为” OR “心理咨询” OR “呼吸训练” OR “冥想” OR “正念” OR “肌肉” OR “太极” OR “心理动力” OR “睡眠教育” OR “睡眠卫生” OR “放松疗法” OR “数字疗法” OR “应用程序” OR “脱敏治疗” OR “家庭治疗” OR “精神分析” OR “中医” OR “中药” OR “按摩” OR “推拿” OR “针灸”）AND（主题: “随机对照” OR “随机”） |
| CNKI=China National Knowledge Infrastructure, ICTRP=International Clinical Trials Registry Platform | |

*Supplemental figure 1 - Funnel plot with trim and fill adjustment*

*Supplemental figure 2 - Meta-regression analysis (age)*

*Supplemental figure 3 - Meta-regression analysis (sample size)*

*Supplemental figure 4* *- Meta-regression analysis (% male)*

*Supplemental figure 5 – Meta-regression analysis (% depression at baseline)*

*Supplemental figure 6* *- Meta-regression analysis (duration of intervention)*
